# Development of the perceived AI–physician consistency and trust scale for cardiology: a Delphi-based study

**DOI:** 10.3389/fdgth.2026.1880574

**Published:** 2026-09-11

**Authors:** Yun Yan, Zhiping Wang, Xueting Wang, Yuxia Zhang, Ping Zhang

**Affiliations:** Department of Intensive Care Unit, Nantong Fourth People’s Hospital, Nantong, Jiangsu, China

**Keywords:** artificial intelligence ethics, cardiovascular disease, critical care, Delphi method, large language models, patient safety, perceived consistency, trust measurement

## Abstract

**Background:**

Large language models (LLMs) have demonstrated considerable potential in cardiovascular diagnostic assistance and clinical decision support. A critical safety-related question, however, remains poorly characterised: how patients subjectively perceive the consistency between artificial intelligence (AI) and physician diagnoses, and how they redistribute trust between the two when their outputs diverge. The costs of miscalibrated trust are ultimately borne downstream by emergency and intensive care services, a pathway typified by decompensating heart failure, in which algorithm-reassured delay converts an outpatient consultation into an intensive care admission. Recurrent clinical antecedents include patient anchoring on the most benign diagnosis offered by AI, and consultation-room friction, often compounded by concealed AI use, that quietly erodes adherence to physician advice. No standardised measurement instrument currently addresses this gap. To develop and content-validate an initial item pool for the Perceived AI–Physician Consistency and Trust Scale for Cardiology (PACT) for use in cardiology outpatient settings, as a preliminary step toward a standardised instrument for evaluating the safety of human–machine interaction in cardiovascular digital health.

**Methods:**

We pre-specified four domains: AI–physician perceived consistency, AI competence trust, AI benevolence trust, and physician reference trust. An initial pool of 10 candidate items was generated. Ten experts from cardiology, medical informatics, and nursing psychology participated in three rounds of Delphi consultation; Rounds 1 and 2 screened and revised the item pool, and Round 3 re-administered the full 12-item pool using the same I-CVI and clarity instrument, with particular focus on confirming the three items whose domain assignment or response format had changed after Round 2. Items were screened using the item-level content validity index (I-CVI) and a 5-point language clarity rating, with retention thresholds of I-CVI ≥ 0.78 and mean clarity score ≥ 3.5. Kendall's coefficient of concordance (W) quantified convergence of expert opinion.

**Results:**

Response rates were 100% in all three rounds, and the mean expert authority coefficient was Cr = 0.86. In Round 1, items Q6 and Q8 yielded I-CVIs of 0.70 and mean clarity scores of 3.3 and 3.5 respectively; both were reconstructed after panel deliberation. The remaining eight items had I-CVIs of 0.90–1.00. Two new items were added on the basis of expert recommendations, producing a 12-item revised pool. In Round 2, all 12 items achieved I-CVIs of 0.90–1.00 and mean clarity scores of 4.2–4.9. Kendall's W for item relevance was 0.35 (*P* = 0.002) in Round 1 and 0.41 (*P* < 0.001) in Round 2; both values indicate weak-to-moderate agreement, and because the two rounds evaluated non-identical item sets these coefficients are reported as within-round descriptive summaries rather than as evidence of increasing consensus. A construct-alignment review conducted for this revision reassigned one item (Q7) to a different domain, reclassified one item (Q9) as a supplementary behavioural item excluded from domain scoring, and corrected an inconsistent Methods description of a third (Q3, whose fielded domain was unchanged); the response anchors for Q1 were also corrected to match its item stem. Because these changes post-dated Round 2, the affected items were re-rated in Round 3 against their revised roles: all ten experts participated (response rate 100%), every item attained an I-CVI of 1.00 with mean clarity scores of 4.2–5.0, modified kappa was 1.000 throughout, and S-CVI/Ave and S-CVI/UA were both 1.000 (Round-2 values 0.975 and 0.750). The resulting four-domain structure comprises 11 domain-scored items and one supplementary behavioural item, each supported by expert ratings obtained for the assignment it now carries.

**Conclusions:**

The three-round Delphi process produced a content-valid 12-item pool (PACT-12) organised into four theoretically derived domains, with satisfactory expert consensus on item relevance and clarity. These results reflect content validation only; patient cognitive interviews, exploratory factor analysis, reliability testing, and criterion-related validation are required before PACT-12 can be regarded as a psychometrically validated or clinically deployable instrument for evaluating patient-side trust shifts and associated safety risks in AI-assisted cardiovascular decision-making.

## 1 Introduction

Large language models (LLMs) are demonstrating increasingly mature performance in diagnostic assistance and clinical decision support within cardiology. Sudri et al. reported that ChatGPT-4 achieved an overall accuracy of 0.82 and a Cohen's *κ* of 0.59 against multidisciplinary heart-team decisions for coronary revascularisation (1). In emergency acuity assessment, Williams et al., drawing on a cohort of 251,401 adult emergency visits, demonstrated that a LLM classified acuity pairs with 89% accuracy, a level comparable to human emergency physicians (2). Masanneck et al. further showed that GPT-4-based ChatGPT and untrained physicians both achieved substantially higher triage concordance than GPT-3.5 (3).

The stability and safety of LLMs in real-world clinical use cannot, however, be assumed. A stress-test by Franc et al. revealed that ChatGPT's overall triage accuracy under the Canadian Triage and Acuity Scale (CTAS) was only 47.5%, with an over-triage rate of 38.7% and an under-triage rate of 13.7% (4). In a cardiology-specific context, Heston and Lewis found that although ChatGPT-4's mean risk stratification for acute non-traumatic chest pain correlated highly with established TIMI and HEART scores (r = 0.898–0.928), its repeated outputs for identical cases exhibited marked dispersion (5). A single query could therefore yield radically different risk classifications. The body of evidence on the objective diagnostic concordance of LLMs is growing, yet a critical translational question remains unanswered: how do patients subjectively perceive AI–physician concordance, and how do they apportion trust when the two sources disagree? There is currently no standardised instrument for measuring this.

The phenomenon of patients arriving at cardiology clinics carrying AI pre-consultation outputs is becoming commonplace. Armbruster et al.'s cross-sectional study highlighted a striking asymmetry: in blinded patient evaluations, ChatGPT scored higher than physicians on empathy (4.18 versus 2.7), yet patients were unable to identify potentially harmful advice (6). In high-stakes presentations such as acute coronary syndrome or acute decompensated heart failure, an AI that provides reassuring output while missing a life-threatening diagnosis may be perceived by the patient as both benevolent and consistent with expectations, potentially producing catastrophic delays in care-seeking. The reverse is equally problematic: a patient who over-trusts AI and perceives its recommendations as inconsistent with the physician's guidance may refuse evidence-based treatment. Both scenarios represent safety failures, and neither can be detected without measuring subjective patient perception.

This concern is sharpened by the research team's clinical base in intensive care. Over the past year, the authors have repeatedly encountered a recurrent admission pattern: patients with decompensated heart failure whose warning symptoms (exertional chest tightness, orthopnoea and paroxysmal nocturnal dyspnoea, palpitations) had been present for days to weeks before deterioration, and whose families report that a LLMs consulted at home had attributed the symptoms to gastro-oesophageal reflux, menopausal syndrome, or neurasthenia and advised rest. A complementary pattern is visible at the other end of the same pathway: patients who did present to the cardiology clinic, but who kept the AI transcript concealed for fear of censure, experienced a time-pressured consultation, read the physician's rapid heuristic exclusion as dismissiveness, and left declining the recommended cardiac workup. In such cases, the AI transcript that never left the patient's pocket and the investigation that was never ordered arrive in the intensive care unit (ICU) together. These are unsystematic clinical observations rather than study data, but they define precisely the phenomenon this instrument is intended to render measurable: the consequences of failed early recognition are managed not in the outpatient clinic where the trust failure originates, but in the ICU where its costs are paid. Heart failure decompensation illustrates the mechanism with particular clarity: unlike hyperacute events, it typically evolves over days, precisely the window in which patients consult AI repeatedly and in which miscalibrated reassurance can convert a ward admission into an intensive care one.

The choice of cardiology as the pilot setting follows directly from this vantage point. Transfers from cardiology have consistently ranked first among sources of admission to the authors' ICU, and cardiovascular disease combines three features that make it the least forgiving arena for pre-consultation trust failure: highly non-specific symptoms, a long compensated phase followed by abrupt decompensation, and narrow therapeutic time windows. The presentations most readily misattributed by AI to digestive or functional disorders (chest pain, chest tightness, palpitations) are precisely those that may precede sudden cardiac deterioration. From a critical care standpoint, an instrument that measures patient-side trust at the pre-consultation stage therefore functions as an upstream safety measure for a downstream problem.

Patient acceptance of AI is systematically lower than acceptance of human physicians, even when clinical performance is equivalent. Longoni et al. attribute this to uniqueness neglect, the belief that AI, operating through standardised algorithms, cannot account for an individual's distinctive clinical circumstances (7). Triberti et al.'s “third-wheel” framework characterises how AI interposed in clinical encounters can generate decision paralysis, symptom misclassification, and confusion about professional responsibility (8). Hatherley has argued on philosophical grounds that AI systems may warrant reliance but not trust, because they lack the benevolent motivation and moral responsibility that trust properly requires (9). Fügener et al. contribute an empirical dimension, showing that human metacognitive deficits constrain collaboration with AI and that effective joint decision-making requires structures that explicitly preserve the autonomy of both parties (10). Taken together, these accounts point to the same gap: the patient's subjective trust architecture in the presence of AI has not been operationalised, and without a validated measure it cannot be studied or managed.

Synthesising these clinical observations with the literature above, we propose two working constructs that describe how pre-consultation AI use translates into delayed or refused care, and which the present instrument is intended to render measurable. The first is false-reassurance anchoring: LLMs typically return a ranked differential spanning benign to serious causes, and patients, following a familiar psychological defence, anchor on the most benign candidate offered (intercostal neuralgia rather than angina, reflux rather than heart failure), which then rationalises continued observation at home. The second is epistemological friction with consequent adherence rupture: a patient who arrives holding an AI-generated panoramic risk map confronts, within a consultation of a few minutes, a physician reasoning by rapid empirical exclusion; the physician's diagnostic shortcuts read to the patient as dismissiveness, the patient conceals the AI consultation for fear of censure, and disagreement that is never voiced becomes non-adherence that is never explained, in the form of declined admission or refused investigation. Nor does the trust process end at the consultation-room door: patients increasingly subject the physician's plan to a post-consultation shadow audit by the same AI, on which adherence to medication and investigation may ultimately turn. These mechanisms jointly motivate a measurement approach that treats trust in AI and trust in the physician not as two separate quantities but as a comparative, potentially competing pair evaluated against the perceived consistency of the two accounts.

Mature instruments exist for measuring patients' trust in physicians. The Trust in Physician Scale (TPS), developed by Anderson and Dedrick (11), remains the most widely used, but it does not accommodate the comparative structure required when patients must simultaneously appraise an AI output and a physician's recommendation. The present study therefore draws on Mayer et al.'s integrative trust model (12) and Festinger's cognitive dissonance theory (13) to develop the Perceived AI–Physician Consistency and Trust Scale for Cardiology (PACT), and tests its content validity through three rounds of Delphi consultation. The aim is to provide a psychometrically grounded instrument for evaluating patient-side safety risks associated with LLM-assisted cardiovascular pre-consultation.

## 2 Materials and methods

### 2.1 Theoretical framework and initial item-pool construction

Scale construction began with Mayer et al.'s integrative trust model (12), which identifies three antecedents of interpersonal trust: ability, benevolence, and integrity. These dimensions were transposed to the patient–LLM context by adding a fourth domain, perceived consistency, grounded in Festinger's cognitive dissonance theory (13). The central proposition from dissonance theory is that when an individual encounters conflicting cognitions, the resulting psychological discomfort motivates reattribution of beliefs; in the present context, a perceived discrepancy between AI and physician output can prompt patients to recalibrate trust in one or both sources. A fifth element, physician reference trust, was introduced to allow the patient's confidence in the physician to serve as an explicit comparator rather than remaining implicit. The four pre-specified domains were therefore: AI–physician perceived consistency, AI competence trust, AI benevolence trust, and physician reference trust.

A core working group based in the ICU of the study hospital, comprising two cardiology chief physicians, one nursing psychology professor with cardiovascular specialisation, and one medical informatics researcher, generated an initial 10-item pool (Q1–Q10) through literature review, the unsystematic clinical observations described in Section 1, and structured clinical vignette discussion (see Supplementary Table S1). Databases searched were PubMed, Web of Science, and CNKI, using the terms “large language model” OR “AI,” combined with “trust,” “patient–physician,” and “cardiology.” Items were fielded on a 5-point Likert response format (1 = strongly disagree, 5 = strongly agree), with Q2 reverse-scored and Q9 treated separately. Two response-format problems were identified during revision and corrected. Q1 is phrased as a degree-of-overlap question rather than an agreement statement, yet was fielded with agree–disagree anchors; it is now assigned an extent-anchored 5-point scale (1 = no overlap at all, 5 = complete overlap) that matches its stem. Q9 is a factual disclosure question and is now recorded in binary (yes/no) form, reported descriptively and excluded from Likert-based domain scoring. Both corrections were introduced after Round 2 and were subsequently rated by the expert panel in Round 3 (Section 3.4), which confirmed them. As fielded, item Q1 was assigned to perceived consistency; Q2 and Q4 to AI competence trust; Q3, Q7, and Q9 to AI benevolence trust; and Q5, Q6, Q8, and Q10 to physician reference trust (Supplementary Table S1). A subsequent construct-alignment review undertaken during manuscript revision (Section 3.4) determined that the domain placement of Q7 and Q9 required correction independent of their Delphi relevance and clarity ratings; the corrected domain assignments were submitted to a third, targeted confirmation round (Section 3.4) and are presented in Table 4.

### 2.2 Delphi expert consultation

#### 2.2.1 Expert selection

Ten experts with recognised standing in cardiology, cardiovascular epidemiology, medical informatics, and nursing psychology were recruited by purposive sampling. This sample size meets the methodological requirement articulated by Polit et al.: with ten or more raters, an item-level content validity index (I-CVI) threshold of 0.78 provides satisfactory content validity after correction for chance agreement (14). Inclusion criteria were: (i) deputy senior professional title or above, or equivalent academic standing; (ii) at least ten years of relevant professional experience; (iii) prior research engagement with cardiovascular AI-assisted care or instrument development; and (iv) willingness to complete the scheduled consultation rounds. The ten enrolled experts (E01–E10) comprised four with full senior titles, four with deputy senior titles, and two at the intermediate level; mean professional experience was 18.6 ± 5.4 years. Disciplinary representation was four from cardiology, two from medical informatics, two from nursing psychology, and two from cardiovascular epidemiology.

#### 2.2.2 Consultation instruments and procedure

The consultation questionnaire comprised three parts: (i) a cover letter describing the study background, accompanied by simulated AI pre-consultation vignettes for the three index presentations (chest pain, palpitations, and dyspnoea); (ii) an item-rating form on which experts independently scored each item for relevance to its assigned domain (1 = irrelevant, 2 = weakly relevant, 3 = quite relevant, 4 = highly relevant) and for language clarity (1 = very unclear, 5 = very clear), with an open column for textual suggestions; and (iii) a self-assessment form for expert background and authority, capturing familiarity with the subject matter (Cs) and the basis for judgement (Ca).

Round 1 was conducted in October 2025. After collection, the I-CVI for each item (the proportion of experts assigning a relevance score of 3 or 4) and the mean clarity score were computed. The pre-specified retention thresholds were I-CVI ≥ 0.78 and mean clarity ≥ 3.5 (14). Items not meeting both criteria were revised or replaced on the basis of expert comments. Round 2 was conducted in December 2025; Round-1 results, including item-level I-CVIs, mean clarity scores, and anonymised expert comments, were returned to the same panel for re-evaluation of the revised items. Round 3 was conducted in August 2026 as a targeted confirmation round. Its scope differed from that of Rounds 1 and 2: rather than screening the item pool afresh, it asked the panel to adjudicate those changes that had been introduced after Round 2 closed and that consequently carried no expert endorsement. The same ten experts were re-invited and all participated. Using the same I-CVI and 5-point clarity instrument as Rounds 1 and 2, experts rated the relevance and clarity of all 12 item against the domain and response format each item now occupies, which for nine items was unchanged from Round 2 and for three items reflected the revision under review: Q7 under Physician Reference Trust, Q9 in its binary behavioural form, and Q1 under its corrected extent-anchored anchors; Round 3 did not include a separate domain-level content-coverage instrument; whether the four domains are adequately and separably covered by their constituent items is an empirical question left to the factor-analytic work described in Section 4.5, not a judgement rendered by Round 3 item ratings. Response rate was 100% in all three rounds. The study conformed to the methodological reporting standards for Delphi research recommended by Diamond et al. (15).

To guard against dominance and conformity effects, experts rated items independently in every round without communicating with one another or with the research team beyond the structured questionnaire; only anonymised summary statistics and anonymised free-text comments were circulated to the panel between rounds, and no expert was informed of any other panellist's identity or individual ratings. Open-ended comments were independently screened by two members of the core working group, grouped thematically (wording ambiguity, domain misalignment, response-format concerns), and used to draft item revisions; disagreements in interpretation were resolved by discussion with a third working-group member before revised items were returned to the panel. The Delphi process was pre-specified to terminate once all items met the retention thresholds (I-CVI ≥ 0.78, mean clarity ≥ 3.5) and item-level indicators showed no further material change between consecutive rounds, up to a maximum of three rounds. These conditions were satisfied for the screened item pool after Round 2. A third round was subsequently convened for the distinct purpose set out above, confirming the post-Round-2 structural changes rather than re-screening the pool, and consultation closed thereafter.

#### 2.2.3 Statistical analysis

Data were analysed in Excel 2021 and STATA 16.0. The expert authority coefficient was calculated as Cr = (Cs + Ca)/2. At the item level, I-CVI and mean clarity were reported for each round. Because I-CVI does not correct for the possibility of chance agreement, a modified kappa was also computed for each item as k* = (I-CVI−p_c_)/(1−p_c_), where pc = [N!/(A!(N−A)!)] × 0.5^N^, N is the number of experts and A the number rating the item as relevant; values above 0.74 denote excellent chance-corrected agreement. Scale-level content validity was summarised in two complementary forms: S-CVI/Ave, the average of the item-level indices, and S-CVI/UA, the proportion of items endorsed by every expert, the latter being the more stringent criterion. Convergence of expert opinion across rounds was assessed with Kendall's coefficient of concordance (W), evaluated by *χ*^2^ test. A two-sided significance threshold of *P* < 0.05 was applied throughout.

### 2.3 Ethics approval

The study was approved by the Institutional Ethics Committee of Nantong Fourth People's Hospital, Jiangsu, China (approval number: 2025-KD037). Written informed consent was obtained from all ten participating experts prior to enrolment. All procedures conformed to the ethical standards of the Declaration of Helsinki and relevant institutional guidelines.

## 3 Results

### 3.1 First-round Delphi consultation

All ten experts returned valid questionnaires, giving a positivity coefficient of 100% and a mean authority coefficient of Cr = 0.86 (familiarity Cs = 0.85; judgmental basis Ca = 0.87). I-CVIs and mean clarity scores for the ten initial items are summarised in Table 1. Item Q6 (“I firmly believe that the physician's clinical decisions are entirely free of economic or convenience considerations, motivated solely by curing my illness”) and item Q8 (“If the AI tells you this is typical stable angina and the situation is not serious at present, while the outpatient physician says there is some risk and further evaluation is needed, which way does your decisional balance lean?”) both yielded I-CVIs of 0.70 and mean clarity scores of 3.3 and 3.5 respectively, falling below both retention thresholds. The remaining eight items had I-CVIs of 0.90–1.00 and mean clarity scores of 4.0–4.9.

**Table 1:** Item evaluation indicators, round 1 Delphi consultation.

No.	Item content (keywords)	I-CVI	Mean clarity	Decision
Q1	Diagnostic perceived consistency	1.00	4.5	Retain
Q2	AI capacity to capture cardiac symptoms	0.90	4.2	Retain
Q3	AI prioritising patient safety	1.00	4.4	Retain
Q4	Consulting AI first for chest discomfort	0.90	4.0	Retain
Q5	Physician grasps symptomatic key	1.00	4.6	Retain
Q6	Physician decisions free of extraneous motives	0.70	3.3	Reconstruct
Q7	Physician first for cardiac problems	1.00	4.9	Retain
Q8	Inclination under inconsistency	0.70	3.5	Reconstruct
Q9	Disclosing AI consultation to physician	1.00	4.6	Retain
Q10	Attribution of anxiety relief	0.90	4.1	Retain

I-CVI, item-level content validity index. Retention thresholds: I-CVI ≥ 0.78 and mean clarity ≥ 3.5.

Expert commentary on Q6 centred on the problem that patients cannot reliably gauge whether extraneous considerations influenced clinical decisions; the phrasing was judged to carry high social-desirability loading and to risk a severe ceiling effect. Reviewers recommended rewriting the item as a behaviourally anchored indicator of physician decisional integrity. Criticism of Q8 focused on the vagueness of “mild inconsistency,” which failed to isolate the safety-critical scenario in which an AI provides a falsely reassuring output while the physician recommends investigation. Multiple experts requested that the item be reframed around a concrete high-risk conflict, specifically the contrast between an AI recommending observation and a physician requesting coronary imaging, so that the item could capture the trust shifts that precipitate treatment delay.

Following these recommendations, Q6 and Q8 were reconstructed, and minor wording revisions were made to Q2, Q4, and Q10. To reinforce the AI benevolence trust and physician reference trust domains, two new items were added: Q11, addressing the patient's protective attribution when AI insists on care-seeking, and Q12, capturing ultimate trust in the physician when clinical reasoning transcends raw data. The revised 12-item questionnaire was submitted for Round 2, as shown in Supplementary Table S2.

### 3.2 Second-round Delphi consultation

All ten original experts participated in Round 2 (response rate 100%; mean authority coefficient Cr = 0.86). Results are shown in Table 2. All 12 items attained I-CVIs of 0.90–1.00 and mean clarity scores of 4.2–4.9, comfortably exceeding the pre-specified thresholds. The two reconstructed items achieved the maximum I-CVI of 1.00. Q6 was reformulated as: “I trust that my physician's treatment plan today was determined solely on the basis of my examination results and clinical needs, and was not influenced by any irrelevant factors.” Q8 was recast as: “When the AI tells me ‘it sounds like it is caused by nerves — you can observe for now,’ while the physician says ‘I am a little concerned; let us do a coronary CT scan,’ I believe the physician's decision is the one that is truly acting in my best interest.” Both new items, Q11 and Q12, achieved I-CVIs of 0.90.

**Table 2:** Item evaluation indicators, round 2 Delphi consultation.

No.	Item content (origin)	I-CVI	Mean clarity	Decision
Q1	Diagnostic perceived consistency (retained)	1.00	4.8	Retain
Q2	AI limitation: lack of physical examination (minor edit)	1.00	4.6	Retain
Q3	AI prioritising patient safety (retained verbatim)	1.00	4.7	Retain
Q4	AI capacity: capturing key information (minor edit)	0.90	4.5	Retain
Q5	Physician grasps symptomatic key (retained verbatim)	1.00	4.8	Retain
Q6	Physician decisional integrity (Round-1 reconstruct)	1.00	4.5	Retain
Q7	Physician first for cardiac problems (retained verbatim)	1.00	4.9	Retain
Q8	Preference for physician in high-risk conflict (Round-1 reconstruct)	1.00	4.4	Retain
Q9	Disclosing AI consultation to physician (retained)	1.00	4.7	Retain
Q10	Attribution of anxiety relief (minor edit)	1.00	4.5	Retain
Q11	AI benevolence: protective insistence on care (new)	0.90	4.2	Retain
Q12	Ultimate physician trust: beyond data (new)	0.90	4.3	Retain

Round-1 reconstructed items Q6 and Q8 each achieved I-CVI, 1.00 after revision. New items Q11 and Q12 reinforce the AI-benevolence-trust and physician-reference-trust domains respectively.

### 3.3 Agreement among experts

In Round 1, Kendall's W for relevance ratings across all items was 0.35 (*χ*^2^ = 31.5, *P* = 0.002), and for clarity ratings W = 0.34 (*χ*^2^ = 30.6, *P* = 0.003). In Round 2, W for relevance was 0.41 (*χ*^2^ = 44.3, *P* < 0.001) and for clarity ratings W = 0.34 (*χ*^2^ = 37.5, *P* < 0.001). By conventional benchmarks, values in this range indicate weak-to-moderate rather than strong agreement, and this should be interpreted cautiously rather than as evidence of strong expert consensus. The Round-2 clarity coefficient is low in absolute terms despite clarity scores that clustered tightly at 4.2–4.9, with a coefficient of variation below 0.10 for every item, and despite I-CVIs of 0.90–1.00 across the 12 items (Table 2). These observations are not in tension. I-CVI and mean clarity summarise the level of expert endorsement, whereas Kendall's W summarises agreement in the ordinal ranking of items across raters and is mechanically attenuated when scores cluster near the ceiling, since near-uniform high ratings generate extensive tied ranks. A low W under ceiling conditions therefore indicates that experts did not rank the items in the same order, not that they disagreed about their adequacy. The Round-1 and Round-2 relevance coefficients should not be read as documenting an increase in consensus. Round 1 W was computed over 10 items and Round 2 W over 12 items, two of which (Q6, Q8) were substantially reworded and two (Q11, Q12) newly introduced between rounds, so the two coefficients were not computed on an identical, unaltered item set. Kendall's W is also a function of the number of items and raters, and no formal significance test exists for the difference between two independently computed W coefficients under these conditions; we therefore report the Round 1 and Round 2 values as within-round descriptive summaries rather than as evidence of a statistically confirmed increase in consensus across rounds. By the criteria of Diamond et al. (15), the satisfactory I-CVI and clarity thresholds, together with stabilising item wording, provide a reasonable basis for terminating this content-validation stage of consultation; Kendall's W should be reported alongside, but not substituted for, these item-level indicators.

### 3.4 Third-round targeted confirmation

Round 3 re-administered the full 12-item pool to the original panel, using the same I-CVI and clarity instrument as Rounds 1 and 2, with particular focus on three items whose role had changed after Round 2 and therefore carried no expert endorsement in that role: the reassignment of Q7 from AI Benevolence Trust to Physician Reference Trust, the reclassification of Q9 as a binary supplementary behavioural item, and the correction of Q1's response anchors. All ten experts from Rounds 1 and 2 participated (response rate 100%); panel composition was unchanged, and no expert was replaced or added. Item-level results for all 12 items are shown in Table 3.

**Table 3:** Item evaluation indicators, round 3 targeted confirmation, with each item rated against its final assigned domain.

No.	Item content (keywords)	Domain rated against in Round 3	I-CVI	Mean clarity	Decision
Q1	Diagnostic perceived consistency (response anchors corrected)	AI–Physician Perceived Consistency	1.00	5.0	Retain
Q2	AI limitation: lack of physical examination (reverse-scored)	AI Competence Trust	1.00	4.6	Retain
Q3	AI prioritising patient safety	AI Benevolence Trust	1.00	5.0	Retain
Q4	AI capacity: capturing key information	AI Competence Trust	1.00	4.5	Retain
Q5	Physician grasps symptomatic key	Physician Reference Trust	1.00	5.0	Retain
Q6	Physician decisional integrity	Physician Reference Trust	1.00	4.5	Retain
Q7	Physician first for cardiac problems (reassigned)	Physician Reference Trust	1.00	5.0	Retain
Q8	Preference for physician in high-risk conflict	Physician Reference Trust	1.00	4.4	Retain
Q9	Disclosing AI consultation to physician (binary format)	Supplementary behavioural item (not domain-scored)	1.00	5.0	Retain
Q10	Attribution of anxiety relief	Physician Reference Trust	1.00	4.5	Retain
Q11	AI benevolence: protective insistence on care	AI Benevolence Trust	1.00	4.2	Retain
Q12	Ultimate physician trust: beyond data	Physician Reference Trust	1.00	4.3	Retain

Round 3 rated each item against the domain to which it is assigned in the final structure (Table 4), including the reassigned item Q7, the reclassified item Q9, and Q1 under its corrected extent-anchored response format. Retention thresholds were unchanged (I-CVI ≥ 0.78, mean clarity ≥ 3.5). All 12 items attained an I-CVI of 1.00 and a modified kappa of 1.000; S-CVI/Ave and S-CVI/UA were both 1.000.

Every item met both retention thresholds when rated against the domain to which it is assigned in the final structure. All 12 items achieved an I-CVI of 1.00, and mean clarity scores ranged from 4.2 to 5.0. The three items whose roles had changed were endorsed in those revised roles specifically: Q7 achieved an I-CVI of 1.00 with mean clarity 5.0 when rated for relevance to Physician Reference Trust rather than to AI Benevolence Trust; Q9 achieved an I-CVI of 1.00 with mean clarity 5.0 in its binary behavioural form; and Q1 achieved an I-CVI of 1.00 with mean clarity 5.0 under its extent-anchored response format. No new items were introduced in Round 3 and none was removed, so the pool remained at 12 items throughout.

At scale level, S-CVI/Ave was 1.000 and S-CVI/UA, the more stringent index requiring endorsement by every expert, was likewise 1.000; the corresponding Round-2 values on the same 12-item set were 0.975 and 0.750. Chance-corrected agreement was excellent for every item, with a modified kappa of 1.000 throughout, compared with a Round-2 range of 0.899 to 1.000. Twelve of 12 items attained an I-CVI of 1.00, against 9 of 12 in Round 2. Kendall's W in Round 3 was 0.479 for relevance (*χ*^2^ = 52.72, df = 11, *P* < 0.001) and 0.471 for clarity (*χ*^2^ = 51.76, df = 11, *P* < 0.001). These coefficients are reported as within-round descriptive summaries and are not compared with the Round 1 or Round 2 values: comparison with Round 1 is precluded by a different item set, and although Rounds 2 and 3 rated the same 12 items, the rating target changed for the three items whose domain assignment or response format had been revised, which is the substance of what Round 3 was convened to assess. The scale-level indices are, by contrast, computed on an identical item set in both rounds and are reported above alongside their Round-2 counterparts.

Round 3 therefore closes the evidential gap identified during peer review with respect to item-level content validity: each item in Table 4 has now been rated by the expert panel in the role it actually occupies. This item-level confirmation should not be conflated with domain-level structural validation. Round 3 did not include a separate instrument for rating the four domains as a whole, and whether the domains are separable in patient response data remains to be established by the exploratory factor analysis described in Section 4.5.

**Table 4:** Final item-domain-scoring table for PACT-12 (supersedes Section 2.1 and Supplementary Tables 1 and 2; the domain assignments for Q7 and Q9 and the response anchors for Q1 were confirmed in the round 3 targeted consultation, Table 3).

No.	Item content (final wording)	Domain (final)	Coding & scoring	Construct-alignment rationale
Q1	In your view, to what extent does the diagnosis or explanation provided by the AI large language model overlap with what today's outpatient physician told you about your diagnosis?	AI–Physician Perceived Consistency	Extent-anchored 5-point scale (1 = no overlap at all, 5 = complete overlap); higher score = greater perceived consistency. Anchors corrected after Round 2 and confirmed in Round 3	Core consistency item; presently the sole item in this domain (see Section 4.5).
Q2	The AI large language model can only assess my condition based on my written descriptions; it cannot perform auscultation or palpation as a physician can. I recognise that this makes its judgements about cardiac problems subject to certain limitations.	AI Competence Trust	Reverse-scored (transformed score = 6−raw); higher transformed score = greater AI competence trust	Agreement reflects perceived AI limitation, i.e., lower competence trust, so raw responses are reversed before domain aggregation.
Q3	I feel that the AI's recommendations are made from my perspective, with protecting my life and safety as the primary goal.	AI Benevolence Trust	Standard; higher score = greater AI benevolence trust	Concerns the AI's perceived intentions, which defines benevolence rather than diagnostic overlap; the block statement in Section 2.1 describing Q1–Q3 as a consistency set did not match how Q3 was actually fielded (Supplementary Table 1) and has been corrected.
Q4	I believe the AI large language model can help me capture and organise the most critical information about my cardiac symptoms, enabling me to describe my condition more clearly to the physician during the consultation.	AI Competence Trust	Standard; higher score = greater AI competence trust	Assesses perceived AI support for symptom communication, a core competence-trust facet.
Q5	I feel that today's outpatient physician fully understood the symptoms I described and identified the key issues.	Physician Reference Trust	Standard; higher score = greater physician reference trust	Concerns the physician's, not the AI's, perceived competence; belongs with the physician-side comparator domain rather than AI Competence Trust as previously stated in Section 2.1.
Q6	I trust that my physician's treatment plan today was determined solely on the basis of my examination results and clinical needs, and was not influenced by any irrelevant factors.	Physician Reference Trust	Standard; higher score = greater physician reference trust	Assesses physician decisional integrity; behaviourally anchored after Round-1 reconstruction (I-CVI improved from 0.70 to 1.00). Content concerns the physician, not AI competence.
Q7	When it comes to cardiac problems, I still place the physician's diagnosis in an irreplaceable first position.	Physician Reference Trust	Standard; higher score = greater physician reference trust	Expresses the patient's ranking of physician above AI (physician primacy), not a belief about the AI's benevolent intent. Fielded in Rounds 1 and 2 under AI Benevolence Trust; reassigned following construct-alignment review and confirmed in Round 3, where it was rated for relevance to Physician Reference Trust (I-CVI = 1.00, mean clarity 5.0).
Q8	When the AI tells me “it sounds like it is caused by nerves — you can observe for now,” while the physician says “I am a little concerned; let us do a coronary CT scan,” I believe the physician's decision is the one that is truly acting in my best interest.	Physician Reference Trust	Standard; higher score = greater physician reference trust	Assesses trust allocation under a concrete high-risk AI–physician conflict; behaviourally anchored after Round-1 reconstruction (I-CVI improved from 0.70 to 1.00).
Q9	During this visit, did you proactively inform the physician that you had previously consulted AI? (Yes/No)	Not domain-scored (supplementary behavioural item)	Binary yes/no; reported descriptively and excluded from domain-score computation	A factual disclosure behaviour rather than a graded attitude; cannot be meaningfully scored on a 5-point agreement scale. Retained as a descriptive corroborating item rather than forced into a domain total. Rated in Rounds 1 and 2 as a 5-point attitudinal item; the binary behavioural format adopted here was rated separately in Round 3 (I-CVI = 1.00, mean clarity 5.0). It also directly indexes the concealment behaviour described in Section 1, which renders AI–physician friction invisible to the treating physician.
Q10	After this outpatient visit, my worry about something seriously wrong with my heart has noticeably eased, and the physician's explanation and judgement were more reassuring to me than the AI's response.	Physician Reference Trust	Standard; higher score = greater physician reference trust	Comparative reassurance item explicitly anchoring physician superiority relative to AI in relieving anxiety.
Q11	When the AI repeatedly advises me to “see a doctor as soon as possible,” I believe this reflects the system's genuine concern for my health rather than a default programmatic prompt.	AI Benevolence Trust	Standard; higher score = greater AI benevolence trust	New item added in Round 2 to reinforce AI Benevolence Trust; distinguishes perceived genuine concern from routine automated prompting.
Q12	Even if all my examination results appear to be within normal ranges, as long as today's physician believes further observation or investigation is warranted, I will choose to trust the physician's clinical judgement rather than reassuring myself on the basis of the data alone.	Physician Reference Trust	Standard; higher score = greater physician reference trust	New item added in Round 2 to reinforce Physician Reference Trust; captures ultimate trust in physician judgement when it diverges from raw examination data.

Coding direction indicates how each item's raw 1–5 Likert response (or, for Q9, binary response) should be treated prior to domain-score computation. ’Standard’ items are scored as given (higher raw score = higher construct score); Q2 is reverse-scored (transformed score = 6−raw). Domain scores are computed as the mean of their constituent items' (transformed) scores and are not combined into a single overall PACT-12 total. The items reassigned (Q7), reclassified (Q9), or reformatted (Q1) after Round 2 were rated by the expert panel in the roles shown here during the Round 3 targeted confirmation (Table 3); the assignments in this table are supported by those ratings rather than by the Round 1 and Round 2 values.

### 3.5 Final scale structure

The Round 1 and Round 2 Delphi ratings evaluated each item's relevance to its originally assigned domain and its language clarity; they did not independently adjudicate whether the *a priori* domain assignment itself was conceptually correct. During preparation of this revision, the research team therefore conducted a separate construct-alignment review of all 12 items, re-reading each item's final wording against the definitions of the four theoretical domains independently of its Delphi performance. This review identified one item whose fielded domain was inconsistent with its content (Q7), one item that functions as a behavioural indicator rather than an attitudinal trust item (Q9), and one item whose fielded domain was in fact correct but had been described inconsistently in the Methods (Q3). A response-format mismatch affecting Q1 was identified at the same time.

Q7 (“When it comes to cardiac problems, I still place the physician's diagnosis in an irreplaceable first position”) was fielded throughout both Delphi rounds under AI Benevolence Trust, but its content expresses the patient's ranking of the physician above AI rather than any belief about the AI's intentions; it has therefore been reassigned to Physician Reference Trust. Q9 (“During this visit, did you proactively inform the physician that you had previously consulted AI?”) is a factual disclosure question rather than an attitudinal statement and cannot be meaningfully scored on a 5-point agreement scale; it is retained as a supplementary behavioural item, reported descriptively, and excluded from domain-score computation. Q3 was fielded, and is retained, under AI Benevolence Trust; the block statement in Section 2.1 describing Q1–Q3 as assigned to perceived consistency reflected only the initial working hypothesis and did not match the domain under which Q3 was actually fielded (Supplementary Table S1). This has been corrected in Section 2.1 of this revision.

Table 4 presents the single, authoritative item-domain-scoring table for PACT-12, superseding all earlier domain descriptions in this manuscript and its supplement. The evidential basis for each assignment warrants explicit statement. In Rounds 1 and 2 the panel rated Q7 for its relevance to AI Benevolence Trust, the domain under which it was then fielded, and rated Q9 as a 5-point attitudinal item; the I-CVI values in Tables 1 and 2 therefore certify those items in their original roles only and cannot be carried over to their revised roles. This is precisely the gap that Round 3 was designed to close. In that round all 12 items, including each reassigned or reformatted item, were rated against the domain and response format each now occupies (Section 3.4, Table 3). Every assignment shown in Table 4 therefore rests on expert ratings obtained for that assignment, not inherited from a superseded one; the adequacy of the four-domain structure taken as a whole is a separate, unresolved question addressed in Section 4.5.

Of the twelve items, eleven are domain-scored across four domains: AI–Physician Perceived Consistency (Q1; one item), AI Competence Trust (Q2, Q4; two items, Q2 reverse-scored), AI Benevolence Trust (Q3, Q11; two items), and Physician Reference Trust (Q5, Q6, Q7, Q8, Q10, Q12; six items). Q9 is retained as a supplementary, non-domain-scored behavioural indicator, reported descriptively (e.g., proportion of patients disclosing prior AI consultation). Higher domain scores indicate greater perceived AI–physician consistency, greater AI competence trust, greater AI benevolence trust, or greater physician reference trust, respectively. Because PACT-12 is designed to detect divergence and redistribution of trust between AI and physician rather than a single undifferentiated construct, the four domain scores should be reported and interpreted separately; they should not be summed into a single total PACT-12 score. The pronounced asymmetry in item allocation across domains, together with the single-item consistency domain, is a substantive limitation of the current pool and is addressed further in Section 4.5; expansion of the Perceived Consistency and AI Competence Trust domains is a priority for the planned pilot validation study.

## 4 Discussion

The present study used a three-round Delphi process to develop PACT-12, a content-validated 12-item pool (11 domain-scored items across four domains, plus one supplementary behavioural item) for measuring perceived AI–physician diagnostic consistency and the distribution of trust between AI and physician sources in cardiology pre-consultation contexts. The scale drew on Mayer et al.'s integrative trust model (12) and Festinger's cognitive dissonance theory (13), and was designed to capture a layer of patient cognition that existing physician-trust instruments do not address. Content validity indices (I-CVI and mean clarity) were uniformly high across all three rounds, and item wording stabilised after Round-1 revisions; however, Kendall's W remained in the weak-to-moderate range throughout, so this stabilisation should be read as satisfactory content validation rather than strong expert consensus, and none of the findings below should be read as evidence of a psychometrically finalised instrument.

The working constructs proposed in Section 1 are deliberately traceable at item level. Q8 operationalises false-reassurance anchoring in miniature: the AI's benign attribution (“it sounds like it is caused by nerves”) set against the physician's concern and request for coronary imaging reproduces exactly the anchor patients are observed to adopt at home. Q1, together with the four-domain architecture as a whole, operationalises epistemological friction by locating the patient's summary judgement of whether the two accounts collide and, when they do, whose account retains authority. Q9, retained as a supplementary behavioural indicator, indexes the concealment behaviour that renders this friction invisible to the treating physician; its binary, non-attitudinal format is a psychometric limitation but a clinical strength, because concealment is precisely the datum the consultation itself cannot capture. Q10's comparative reassurance framing reaches toward the post-consultation phase, in which the physician's plan may be subjected to a shadow audit by the same AI; capturing that phase fully will require the longitudinal extension noted in Section 4.5.

### 4.1 Theoretical appropriateness

Mayer et al.'s tripartite model was originally formulated for organisational contexts, but its constructs map naturally onto the patient's evaluation of any trustee (12). The ability dimension captures whether the patient believes the AI or physician can perform the diagnostic task competently. Benevolence captures whether the patient attributes good intentions to the source, and integrity captures whether the patient trusts that the source's outputs reflect genuine clinical reasoning rather than extraneous factors. The central innovation of PACT is the addition of a perceived-consistency domain, which operationalises the dissonance that arises when the two sources give different signals. According to Festinger's framework (13), this dissonance motivates cognitive reattribution, and it is precisely this reattribution that determines whether the patient follows the physician's recommendation or delays care. No existing instrument captures this dynamic, including the Trust in Physician Scale (11), which was designed for contexts in which AI was not a factor. The physician-reference-trust domain also addresses a gap identified by Lorenzini et al., who argued that the introduction of AI clinical decision support transforms the doctor–patient dyad into a triad, requiring relational instruments that accommodate the contributions of all three parties (16).

### 4.2 Methodological contributions of the Delphi process

The clinical context for which PACT was developed is one in which measurement error carries direct safety implications. An item prone to ceiling effects or social-desirability bias may obscure the very patients who are most at risk of delaying care because they have uncritically accepted an AI reassurance. The Delphi iteration identified and corrected two such items. The original Q6 asked whether the physician's decisions were free of extraneous motives, a formulation that almost any patient would endorse, rendering the item incapable of discrimination. Its replacement anchors the construct in observable clinical behaviour. Q8 underwent an analogous transformation: the original version described a generic inconsistency, whereas the revised version presents the specific conflict between an AI recommending observation and a physician requesting coronary imaging. This scenario is directly relevant to the safety risks documented by Franc et al. (4) and Heston and Lewis (5), and it is precisely the type of situation in which an erroneous trust allocation could precipitate irreversible harm. The capacity to modify items through multiple expert cycles is a recognised strength of the Delphi method for instrument development in clinically sensitive areas (15). This framing, and the acute high-acuity scenarios (for example, acute coronary syndrome or acute decompensated heart failure) invoked in Section 1 to motivate the study, describe why the underlying clinical problem matters; they are drawn from the cited external literature on AI diagnostic behaviour and patient trust, not from any finding generated by PACT-12 itself. Content validity establishes only that the expert panel judged Q8 relevant to, and clearly worded for, this type of conflict; it does not establish that patients' Q8 responses actually track, predict, or discriminate real-world safety outcomes such as delayed presentation or missed high-acuity diagnoses. That inference would require criterion-related and predictive validity work, for example correlating PACT-12 scores with objectively recorded care-seeking behaviour or clinical outcomes in a patient cohort, which has not yet been undertaken and is identified as a priority in Section 4.5.

### 4.3 Comparison with existing instruments

The trust measurement landscape in digital health is evolving, but no validated instrument simultaneously captures the patient's perception of AI–physician concordance and their relative trust in each source under conditions of disagreement. The TPS (11) and its derivatives measure physician trust in isolation. The Theory of Trust and Acceptance of Artificial Intelligence Technology, developed by Stevens and Stetson (17), provides a validated clinician-facing measure of AI-specific trust but was not designed for patients and does not include a physician-reference comparator. Woodcock et al. examined how explanations affect layperson trust in AI symptom checkers (18), finding that explanatory content modestly increased trust but did not address the comparative trust question that arises when AI and physician outputs differ. PACT is the first instrument to operationalise all three elements simultaneously: AI competence, AI benevolence, and physician reference trust, anchored to a perceived-consistency dimension that captures the patient's cognitive response to disagreement. A single reverse-scored item is currently included in the pool (Q2, within AI Competence Trust; Table 4), intended to guard against the acquiescence bias that typically inflates agreement ratings in Likert-format questionnaires. Because the remaining eleven domain-scored items are all positively worded, including those within AI Benevolence Trust and Physician Reference Trust, this protection is only partial; the addition of further negatively worded items across domains is identified as a priority for the next phase of item development in Section 4.5.

### 4.4 Clinical implications

Cai et al. recently demonstrated, using a mediation model and agent-based simulation in a Chinese sample, that higher AI trust was associated with delayed healthcare-seeking, particularly among patients with chronic conditions and frequent AI users (19). Their findings lend prospective relevance to PACT: if a validated instrument can identify patients with inappropriately elevated AI trust or pronounced perceived inconsistency, clinicians could target those patients for structured communication before the trust configuration becomes a safety hazard. Hatherley's philosophical account (9) complicates this picture: even if a physician recognises that a patient trusts AI inappropriately, the conceptual distinction between reliable-but-not-trustworthy and trustworthy in the full interpersonal sense may be difficult to communicate in a clinical encounter. Once validated, the PACT-12 item set could provide a concrete behavioural anchor for that conversation.

Funer and Wiesing argue that AI support tools create a normative tension for physicians, who must balance deference to algorithmic recommendations against the exercise of independent clinical judgement (20). From the patient's side, an analogous tension exists: following AI recommendations may feel rational, given their surface-level confidence and accessibility, yet doing so at the expense of the physician's guidance could compromise care. PACT-12 is intended to surface this tension quantitatively once its psychometric properties have been established, and its physician-reference-trust domain is intended to help reveal cases in which AI-derived reassurance may have displaced rather than supplemented trust in the physician. Singavarapu et al. evaluated four major chatbots on the quality and accuracy of cardiovascular disease information and found wide variation in both understandability and misinformation rates (21). This reinforces the argument that the quality of AI cardiovascular information cannot be assumed, and that instruments measuring patient response to AI outputs in this domain will need the precision that a validated, scenario-anchored scale could provide.

From a critical care perspective, these implications carry particular weight. The three index presentations used in this study's vignettes (chest pain, palpitations, dyspnoea) are precisely those whose missed escalation populates coronary and ICU: the myocardial infarction that presents after hours of AI-reassured observation, and the decompensated heart failure admitted after days of self-managed deterioration, are the same upstream trust failure seen from the intensivist's side of the door. Under-triage of the kind documented by Franc et al., in which 13.7% of presentations received a lower urgency rating than warranted (4), does not remain an outpatient problem: the patients it misses surface as intensive care admissions, later, sicker, and with fewer therapeutic options. Miscalibrated patient-side trust at the pre-consultation stage can therefore be regarded as an upstream determinant of downstream acuity. Once PACT-12 has been validated in patient samples, a line of inquiry of particular interest to intensivists is whether specific trust configurations, for example high AI competence trust combined with low physician reference trust, are associated with longer symptom-to-presentation intervals or higher acuity at admission; such studies would link the construct measured here to the case mix that critical care services ultimately inherit. Any such application presupposes the psychometric validation programme described in Section 4.5 and is not supported by the present content-validation evidence alone.

### 4.5 Limitations and future directions

This study has several important limitations that should guide interpretation of PACT-12 at its current stage of development.

First, the present work provides expert-based content validation only. No patient cognitive interviews, pilot testing, exploratory or confirmatory factor analysis, internal-consistency reliability assessment, test–retest evaluation, or criterion-related validation have yet been conducted. PACT-12 should accordingly be regarded as a preliminary, content-validated item pool rather than a validated, reliable, or clinically deployable scale, and none of the findings reported here should be interpreted as evidence of construct validity, reliability, or clinical utility. This caveat applies with particular force to the high-acuity scenarios, such as acute coronary syndrome and acute decompensated heart failure, used in Section 1 to motivate the study and referenced in Section 4.2 in relation to Q8: expert agreement that an item is relevant to such a scenario is evidence of content validity only, and does not demonstrate that PACT-12 scores can detect, predict, or mitigate real safety outcomes in these presentations. Establishing any such link will require dedicated criterion-related and predictive validity studies against objectively recorded clinical endpoints, for example care-seeking delay or missed high-acuity diagnoses, which is beyond the scope of the present Delphi-based content-validation study and is not claimed here. The same status applies to the mechanistic account offered in Section 1 (false-reassurance anchoring, epistemological friction with adherence rupture, concealment, and post-consultation shadow auditing): it is a motivating hypothesis set derived from unsystematic clinical observation and the cited literature, not a demonstrated causal pathway, and the qualitative companion study described in the final paragraph of this section is designed to examine it.

Second, item generation and evaluation in this study were conducted exclusively by clinical and informatics experts on a ten-member panel that satisfies the methodological minimum for I-CVI-based content validation with correction for chance agreement (14), but included no patients, no patient advocates, no general practitioners working at the front line of AI-assisted presentations, and no critical care physicians independent of the investigator team. No patient contributed to item wording, selection, or interpretation, notwithstanding that PACT-12 is designed to measure patients' subjective trust experiences. This expert-only process is a structural feature of a Delphi-based development phase rather than an incidental gap, and it is the reason patient cognitive interviews and face-validity testing, to confirm that patients interpret each item as intended and that the item set captures the range of patient experience, are treated as a required rather than optional next step before any quantitative pilot validation is undertaken.

Third, although the Round 3 consultation confirmed each item in its final role, this is item-level content validity only. No separate domain-level instrument was administered in any round, and the four domains have not themselves been rated or endorsed as a structure. Content validity of this kind remains expert-referenced judgement about item relevance and wording. It is not evidence of construct validity, and it cannot substitute for the empirical demonstration that the four domains are separable in patient response data. The structure in Table 4 is best understood as a theoretically coherent hypothesis about scale structure, with each constituent item individually content-validated, to be tested rather than assumed in the factor-analytic work described below. A further caveat is that the same ten experts served in all three rounds, so the Round 3 ratings are not independent of the earlier ones: panellists were confirming revisions to items they had themselves helped shape, which may favour endorsement.

Fourth, domain allocation remains uneven, and Round 3 did not alter this. Because that round was convened to adjudicate the post-Round-2 domain and format changes rather than to extend the pool, no items were added, and the distribution is unchanged: AI–Physician Perceived Consistency contains a single item (Q1), AI Competence Trust two (Q2, Q4), AI Benevolence Trust two (Q3, Q11), and Physician Reference Trust six (Q5, Q6, Q7, Q8, Q10, Q12), with Q9 retained outside domain scoring. The confirmation round therefore settled whether each item sits in a defensible domain, but not whether each domain contains enough items to be measured. The psychometric consequences of any residual imbalance constrain the analyses planned below and should be stated plainly. Internal consistency cannot be estimated for a domain represented by a single item, and Cronbach's *α* is a poor choice for two-item domains, where it is known to underestimate reliability and to be difficult to interpret. A domain represented by a single item also cannot emerge as a factor in exploratory factor analysis, so a pool containing one cannot in principle confirm the four-domain structure it proposes. Where domains remain short, further item development to a minimum of three or four items each, undertaken before rather than after quantitative validation, is treated as a prerequisite for meaningful structural and reliability analysis, alongside possible reduction of the Physician Reference Trust set once empirical item-discrimination data are available.

Fifth, as noted in Section 4.3, only one item (Q2) is reverse-scored; the predominance of positively worded items, particularly within AI Benevolence Trust and Physician Reference Trust, leaves the pool only partially protected against acquiescence bias. Additional negatively worded items should be developed and tested during pilot validation.

Sixth, the simulated clinical vignettes were restricted to acute presentations; acute decompensated heart failure presenting as dyspnoea falls within this scope, but applicability to chronic disease management, including the longitudinal follow-up of stable heart failure and anticoagulation monitoring, requires separate evaluation. Seventh, the item set was calibrated to 2025-generation LLMs; given the pace of model development, a periodic review mechanism should be built into the instrument's lifecycle.

The research team plans to enrol approximately 200 cardiology outpatients in a pilot validation study, preceded by the patient cognitive interviews described above in a smaller sample. In parallel, a qualitative companion study is planned: semi-structured exit interviews with cardiology outpatients, conducted immediately after the consultation while recall of the encounter is freshest, tracing how pre-consultation AI outputs shaped disease expectations, which moments within the consultation opened or closed disclosure of AI use, and how post-consultation shadow auditing of the physician's plan influences adherence; its findings will inform item refinement prior to pilot validation and provide qualitative context for interpreting PACT-12 scores. The targeted confirmation round has now been completed (Section 3.4); the pilot will follow any further item development required to bring short domains to a length at which structural and internal-consistency analyses are interpretable. Items will be evaluated by critical-ratio analysis, corrected item–total correlations, and exploratory factor analysis. Reliability statistics will be selected to match domain length rather than applied uniformly: Cronbach's *α*, reported alongside McDonald's *ω*, will be used for any domain containing three or more items; for any domain that remains at two items the Spearman–Brown coefficient will be reported instead, as *α* is biased and poorly interpretable at that length; and for any domain that remains single-item, no internal-consistency coefficient will be computed, with test–retest stability (intraclass correlation coefficient) serving as the only available reliability index. Test–retest reliability will in any case be assessed for all domains and for the supplementary behavioural item. Two subsequent extensions are planned that reflect the research team's critical care setting: adaptation, with separate validation, of the item pool for post-discharge follow-up of cardiac intensive care survivors, notably those with heart failure, a population at high readmission risk in which AI consultation typically continues during recovery; and linkage studies examining whether pre-consultation trust configurations are associated with symptom-to-presentation intervals and acuity at admission. Only after this programme of patient-based psychometric validation has been completed, and if the four-domain structure and item allocation are empirically confirmed or revised accordingly, would it become appropriate to consider PACT-12 for bedside screening or for research into the relationship between AI trust distribution and clinical safety outcomes in cardiovascular digital health.

## 5 Conclusion

The three-round Delphi process produced a 4-domain, content-validated item pool for measuring perceived AI–physician consistency and multidimensional trust in cardiology pre-consultation settings, comprising 11 domain-scored items and one supplementary behavioural item (Q9). Item-level content validity indices and clarity ratings were consistently satisfactory across all three rounds; Kendall's W, however, remained in the weak-to-moderate range and should not be read as evidence of strong overall consensus. A construct-alignment review reassigned one item (Q7), reclassified a second (Q9) as a non-domain-scored behavioural indicator, corrected an inconsistent Methods description of a third (Q3, whose fielded domain was unchanged), and corrected the response anchors of Q1. Because these changes post-dated Round 2 and therefore carried no expert endorsement, a third targeted consultation round was conducted with the same panel, in which all 12 items, including every reassigned or reformatted item, were rated against the role each now occupies. Table 4 accordingly rests on item-level ratings obtained for the assignments it reports. The four-domain structure itself awaits confirmation by the factor-analytic work described in Section 4.5. At this stage, PACT-12 represents a preliminary, content-validated step toward filling a gap in the cardiovascular digital health literature: an eventual tool that captures not only whether patients trust AI but how that trust interacts with, and potentially displaces, trust in the physician. Patient cognitive interviews, exploratory factor analysis, reliability and test–retest evaluation, and criterion-related validation in a clinical sample are required before PACT-12 can be considered a validated measure; that programme of work will also determine whether the current pool should be reduced, whether the present four-domain structure and item allocation are empirically supported, and whether the instrument is sensitive to the trust shifts that the evidence base suggests carry direct implications for patient safety.

## Data Availability

The datasets presented in this article are not readily available because the raw data supporting the conclusions of this paper are held by Nantong Fourth People's Hospital, China. Further access requires institutional approval. Requests to access the datasets should be directed to Zhiping Wang, 18888058897@139.com.
