# Supplementary material for: Development of the perceived AI–physician consistency and trust scale for cardiology: a Delphi-based study
Source: Front Digit Health. 2026 Sep 11;8:1880574. doi: 10.3389/fdgth.2026.1880574 (PMC13612560; doi:10.3389/fdgth.2026.1880574)
Supplement: Supplementary file 1 [file Table1.docx]

**Supplement Material**

**Table 1 Round 1 Expert Consultation Questionnaire (10 items)**

| **No.** | **Item content** | **Domain** | **Decision** |
| --- | --- | --- | --- |
| **Q1** | In your view, to what extent does the diagnosis or explanation provided by the AI large language model overlap with what today's outpatient physician told you about your diagnosis? | AI–Physician Perceived Consistency | **Retain** |
| **Q2** | I believe the AI large language model can accurately capture the cardiac discomfort symptoms I describe (such as chest tightness, irregular heartbeat, and shortness of breath). | AI Competence Trust | **Retain** |
| **Q3** | I feel that the AI's recommendations are made from my perspective, with protecting my life and safety as the primary goal. | AI Benevolence Trust | **Retain** |
| **Q4** | Overall, I am willing to consult AI first next time I experience palpitations or chest tightness before deciding whether to visit hospital. | AI Competence Trust | **Retain** |
| **Q5** | I feel that today's outpatient physician fully understood the symptoms I described and identified the key issues. | Physician Reference Trust | **Retain** |
| **Q6** | [Round-1 original] I firmly believe that the physician's clinical decisions are entirely free of economic or convenience considerations, motivated solely by curing my illness.  Expert concern: Social-desirability loading is extremely high — virtually all patients would agree, producing a severe ceiling effect. Experts recommended reformulation as a behaviourally anchored statement of physician decisional integrity. | Physician Reference Trust | **Reconstruct** |
| **Q7** | When it comes to cardiac problems, I still place the physician's diagnosis in an irreplaceable first position. | AI Benevolence Trust | **Retain** |
| **Q8** | [Round-1 original] If the AI tells you "this is typical stable angina, the situation is not serious at present," while the outpatient physician says "there is some risk, further evaluation is needed" — faced with this mild inconsistency, which way does your decisional balance lean?  Expert concern: The scenario is too generic; "mild inconsistency" fails to isolate the high-risk conflict that characterises cardiology emergencies. The question-and-answer format cannot accommodate a Likert response scale. | Physician Reference Trust | **Reconstruct** |
| **Q9** | During this visit, did you proactively inform the physician that you had previously consulted AI? | AI Benevolence Trust | **Retain** |
| **Q10** | After today's outpatient visit, the burden you had been carrying (the fear of something seriously wrong with your heart) — who was mainly responsible for lifting it? | Physician Reference Trust | **Retain** |

**Table 2 Round 2 Expert Consultation Questionnaire (12 items, with revision notes)**

| **No.** | **Item content (Round-2 revised version)** | **Domain** | **Revision note** |
| --- | --- | --- | --- |
| **Q1** | In your view, to what extent does the diagnosis or explanation provided by the AI large language model overlap with what today's outpatient physician told you about your diagnosis? | AI–Physician Perceived Consistency | Retained verbatim. No changes made. |
| **Q2** | The AI large language model can only assess my condition based on my written descriptions; it cannot perform auscultation or palpation as a physician can. I recognise that this makes its judgements about cardiac problems subject to certain limitations. | AI Competence Trust | Minor wording edit: The original was positively framed around AI's ability to "accurately capture" symptoms, creating a positive response bias and potential ceiling effect. The revised item redirects the construct toward AI's limitation in lacking physical examination capability, improving discriminative power within the AI-competence-trust domain. |
| **Q3** | I feel that the AI's recommendations are made from my perspective, with protecting my life and safety as the primary goal. | AI Benevolence Trust | Retained verbatim. No changes made. |
| **Q4** | I believe the AI large language model can help me capture and organise the most critical information about my cardiac symptoms, enabling me to describe my condition more clearly to the physician during the consultation. | AI Competence Trust | Minor wording edit: The original focused on willingness to "consult AI before deciding to visit hospital," which leans toward behavioural intention and fits less well within the AI-competence-trust domain. The revised item focuses on AI's value in organising symptom information, clarifying the construct alignment and removing any implication that AI might substitute for the decision to seek care. |
| **Q5** | I feel that today's outpatient physician fully understood the symptoms I described and identified the key issues. | Physician Reference Trust | Retained verbatim. No changes made. |
| **Q6** | I trust that my physician's treatment plan today was determined solely on the basis of my examination results and clinical needs, and was not influenced by any irrelevant factors. | Physician Reference Trust | Reconstructed (original I-CVI = 0.70): The original phrasing ("entirely free of economic or convenience considerations") carried extremely high social-desirability loading. The revision replaces the subjective motivation attribution with a behaviourally anchored statement ("based on examination results and clinical needs"), making physician decisional integrity concrete and discriminable. I-CVI improved from 0.70 to 1.00. |
| **Q7** | When it comes to cardiac problems, I still place the physician's diagnosis in an irreplaceable first position. | AI Benevolence Trust | Retained verbatim. No changes made. |
| **Q8** | When the AI tells me "it sounds like it is caused by nerves — you can observe for now," while the physician says "I am a little concerned; let us do a coronary CT scan," I believe the physician's decision is the one that is truly acting in my best interest. | Physician Reference Trust | Reconstructed (original I-CVI = 0.70): The original scenario was too abstract ("mild inconsistency"), and the question-and-answer format was unsuitable for Likert scoring. The revised item presents a concrete high-risk conflict (AI recommends observation vs physician recommends coronary imaging), directly measuring the patient's trust allocation under a clinically meaningful disagreement with implications for care-seeking delay. I-CVI improved from 0.70 to 1.00. |
| **Q9** | During this visit, did you proactively inform the physician that you had previously consulted AI? | AI Benevolence Trust | Retained verbatim. No changes made. |
| **Q10** | After this outpatient visit, my worry about something seriously wrong with my heart has noticeably eased, and the physician's explanation and judgement were more reassuring to me than the AI's response. | Physician Reference Trust | Minor wording edit: The original open-ended "who helped you" question cannot be scored on a Likert scale and presents AI and physician contributions as alternatives without explicit comparison. The revised item is a scorable declarative statement that explicitly anchors physician superiority in anxiety relief, providing directional measurement of physician reference trust. |
| **Q11** (new) | When the AI repeatedly advises me to "see a doctor as soon as possible," I believe this reflects the system's genuine concern for my health rather than a default programmatic prompt. | AI Benevolence Trust | Newly added: Added to reinforce the AI-benevolence-trust domain. This item measures the patient's protective attribution of AI's insistence on care-seeking, distinguishing "programmatic response" from "genuine concern." It helps reveal the deeper psychological structure of AI benevolence trust. |
| **Q12** (new) | Even if all my examination results appear to be within normal ranges, as long as today's physician believes further observation or investigation is warranted, I will choose to trust the physician's clinical judgement rather than reassuring myself on the basis of the data alone. | Physician Reference Trust | Newly added: Added to reinforce the physician-reference-trust domain. This item measures the patient's ultimate trust in the physician's clinical experience when objective data (normal examination findings) and physician judgement (recommendation to investigate further) diverge. This construct is directly linked to treatment adherence and safe care-seeking behaviour. |
